# The Hole and the Whole: Lessons from Manipulation of *Nipbl* Deficiency

**DOI:** 10.1371/journal.pbio.2000494

**Published:** 2016-09-08

**Authors:** Bruce D. Gelb

**Affiliations:** The Mindich Child Health and Development Institute and the Departments of Pediatrics and Genetics and Genomic Sciences, Icahn School of Medicine at Mount Sinai, New York, New York, United States of America

## Abstract

Congenital heart defects (CHDs) affect 2%–3% of newborns and remain challenging clinically. There is an ongoing project to elucidate the causes of CHDs, focusing primarily on genetics as dictated by the epidemiology. In a paper published in this issue, Santos and colleagues describe studies of Cornelia de Lange syndrome-associated secundum atrial septal defects (ASDs) caused by *NIPBL* mutations, undertaken with a targeted trapping allele in mice. They show that *Nipbl* haploinsufficiency in either of two cell populations was sufficient to engender ASDs but that expression solely in either one of those populations was sufficient to rescue them. This work provides novel insights into incomplete penetrance and oligogenic effects underlying CHDs.

The etiology of congenital heart defects (CHDs) is a topic of considerable interest [1]. Clinically, CHDs are the commonest forms of birth defects and, despite remarkable advances in care, remain a leading cause of mortality among birth defects [2]. Biologically, the development of the cardiovascular system is quite complex, with contributions from a variety of progenitor populations—the first heart field, second heart field, cardiac neural crest, and proepicardial organ—as well as interactions with other nearby tissues [3,4]. Elucidating the causes of CHDs has been challenging. Epidemiologic studies have implicated genetic factors as the primary causes [1]. Amongst those, we have long understood that aneuploidies and single-gene traits, mostly syndromic ones, constitute a bit less than 10% of cases. Starting in the 1980s, genetics-first approaches for disease gene discovery have enabled the identification of CHD genes, leveraging families inheriting CHDs in Mendelian fashion. More recently, advances in genomic technologies have enabled major advances in our understanding of CHD etiology. Copy number variations and de novo point and indel mutations each account for approximately 10% of CHDs [5–7]. Thus, in aggregate, nearly one-third of CHDs can be explained genetically, at present.

What, then, of the remaining unexplained 70% of CHD cases? As CHDs are viewed principally as a complex genetic trait (or an extended series of such traits, actually), a leading consideration to account for a substantial proportion of the unknown is that several mutations altering various genes relevant for cardiogenesis combine to result in CHDs. Such an oligogenic model is theoretically sound but practically difficult to validate experimentally.

In the fascinating research study described in this issue of *PLOS Biology*, Rosaysela Santos and her colleagues took a novel approach to understanding CHDs observed in a single-gene trait, Cornelia de Lange syndrome (CdLS), using a sophisticated conditional allele in mice [8]. In so doing, they made novel observations that add further complexity to the way in which we need to think about CHD pathogenesis.

CdLS is a rare genetic disorder that is phenotypically and genetically heterogeneous [9]. Approximately 30% of affected individuals have a CHD, but the types are somewhat variable [10,11]. Pulmonary valve and/or artery abnormalities, ventricular septal defects, and secundum atrial septal defects (ASDs) account for the majority. Genetically, heterozygous mutations in three autosomal genes (*NIPBL*, *RAD21*, and *SMC3*) and hemizygous ones in two genes residing on chromosome X (*HDAC8* and *SMC1A)* account for roughly 70% of cases, with *NIPBL* being predominant (~60% of cases) [9]. *NIPBL* encodes a protein similar to *Drosophila* Nipped-B (so NIPPED-B-LIKE) and associates with and regulates the cohesin complex, which is critical for chromosome segregation during mitosis. Most of the proteins encoded by the other CdLS genes are also components or regulators of cohesin complexes such that the trait is now classified as a cohesinopathy. These complexes, however, have roles in addition to that in mitosis. For the pathogenesis of CdLS, the available evidence suggests that alterations in gene expression, some mediated through cohesin loading and others potentially through binding of NIPBL to active promoters as a transcriptional co-factor, lead to the developmental phenotypes [12]. In support of this, gene expression studies with CdLS animal models and cultured human cells have revealed modest alterations in a substantial number of genes. Thus, CdLS is itself a model of oligogenic effects leading to CHDs.

Central questions about the origins of CHDs in CdLS, ones shared with many other genetic traits with CHDs, include why some affected patients have CHDs while others do not and how the lesion specificity arises. Given the low frequency of the condition and the large number of alleles, those issues are difficult to address in humans due to problems with statistical power. Instead, investigators have focused on modeling CdLS in animals, hoping to recapitulate the disease and then interrogate the outstanding questions. The research groups of the collaborating senior authors for Santos et al., Anne Calof and Arthur Lander, have previously established CdLS animal models in zebrafish and mice [13–15]. Particularly germane here: their previous CdLS mouse model, a global *Nipbl* knock-out allele, exhibited large ASDs in ~30% of affected offspring [15].

In Santos et al. [8], the research team used a targeted trapping allele, Flip-Excision (FlEx) [16], in *Nipbl*. The flexibility engineered into this technology allowed them to conditionally knock out *Nipbl*, similar to a more typical Cre-Lox strategy, but also to conditionally express wild-type *Nipbl* at physiologic levels in the context of a global *Nipbl* knock-out. Using Cre drivers that are relevant for various cardiovascular progenitor populations contributing to cardiogenesis, the authors leveraged the conditional knock-out capability to isolate the relevant contributors to the ASD phenotype in CdLS mice. This is a widely used approach and, in this instance, showed that loss of *Nipbl* in the developing myocardium or endoderm phenocopied global loss of *Nipbl* with an ASD prevalence of ~30%. In contrast, loss of *Nipbl* in the developing cardiac neural crest did not engender any ASDs. Of note, the authors did not report the use of a second heart field-specific Cre. While knowing that derivatives of two cell populations are germane to CdLS-related ASDs is interesting enough, the established contribution of second heart field derivatives to atrial formation makes them particularly relevant to this cardiac phenotype and might be worth exploring at some juncture.

Next, the authors used the same Cre drivers to explore whether conditional expression of *Nipbl* on the global *Nipbl* knock out background rescued the ASD [8]. Here, the fireworks begin (it is July 4 as I write this, so why not!). Conditional expression of *Nipbl* in the developing myocardium or in the endoderm (and its derivatives) was able to rescue from ASD. These were, of course, mind-bending results! Loss of *Nipbl* in only the developing myocardium was sufficient to engender ASDs but its restoration in only the developing endoderm (i.e., in the presence of *Nipbl* deficiency in the developing myocardium) was sufficient to rescue ASDs. And vice versa. Since both sets of results entail *Nipbl* expression in one tissue but not the other and with opposite outcomes, the authors logically turned to consider what had changed elsewhere: the presence or absence of *Nipbl* expression in other tissues. They posit that alterations in body size, including heart size, may be crucial. That hypothesis is appealing and provocative but will require testing. Meanwhile, there are other, equally interesting possibilities that could be explored: interactions with other cardiac progenitor populations or secreted factors from non-cardiac tissues being two that readily come to mind.

The authors of Santos et al. undertook one last set of experiments to address the incomplete penetrance of ASD in their CdLS mouse model [8]. Knowing the role of *NKX2*.*5* in cardiac development and having established that its expression is modestly reduced in the *Nipbl* knock-out mouse model, they developed a genetically sensitized model by generating mice doubly heterozygous for *Nipbl* and *Nkx2*.*5* deficiency globally. This genetic combination increased CHD prevalence to over 80%, and included isolated ASDs but also more complex forms of CHDs such as truncus arteriosus. Extrapolating this to study the diverse cardiac phenotypes in patients with CdLS, including those with normal hearts, would seem possible, albeit potentially difficult to adequately statistically power.

The possible relevance of gene–gene and gene–environment interactions for the pathogenesis of CHD has been with us for some time [17]. Indeed, it is the basis for James Nora’s multifactorial inheritance model of CHD, which remains as the best available for the field [18,19]. In addition to the results of this study, which bring potential issues of the genotype status of non-cardiac tissues in the developing fetus to the fore, we are also recognizing potential relevance of fetal-maternal gene and environmental influences. For instance, Patrick Jay’s research team recently explored CHD in *Nkx2*.*5* haploinsufficient mice, focusing more on their ventricular septal defects than ASDs [20]. Specifically, they examined the role of increasing maternal age as a risk factor for CHD. Using a clever strategy with reciprocal ovarian transplantation, they isolated advancing age of the mother but not her ovaries as a risk factor driving ventricular septal defect prevalence in *Nkx2*.*5* haploinsufficient offspring. Moreover, they showed that an exercise regimen begun earlier in life mitigated that risk, through an as-yet-unidentified mechanism. Taken with the results of Santos and colleagues, it becomes clear that a full understanding of the etiology of CHD will require a holistic approach to complete this project successfully. Sophisticated animal modeling studies will need to be paired with careful epidemiology that will consider genetic and environmental factors acting upon the fetus and his or her expectant host. There is surely a whole lot more to do before we completely understand the origins of even simple holes in the heart.

## Abbreviations

ASD: atrial septal defect
CdLS: Cornelia de Lange syndrome
CHD: Congenital heart defect
FlEx: Flip-Excision
